# The expression and function of TRPV4 channels in primate retinal ganglion cells and bipolar cells

**DOI:** 10.1038/s41419-019-1576-3

**Published:** 2019-05-07

**Authors:** Fan Gao, Zhuo Yang, Roy A. Jacoby, Samuel M. Wu, Ji-Jie Pang

**Affiliations:** Department of Ophthalmology, Baylor College of Medicine, One Baylor Plaza, NC 205, Houston, TX 77030 USA

**Keywords:** Cell death in the nervous system, Neurodegenerative diseases

## Abstract

The transient receptor potential vanilloid 4 (TRPV4) channel may be opened by mechanical stimuli to mediate Ca^2+^ and Na^+^ influxes, and it has been suggested to mediate glaucoma retinopathy. However, it has been mostly unclear how TRPV4 activities affect the function of primate retinal ganglion cells (RGCs). We studied RGCs and bipolar cells (BCs) in the peripheral retina of the old-world primate using whole-cell current-clamp and voltage-clamp recordings, immunomarkers and confocal microscopy. RGCs were distinguished from displaced amacrine cells (ACs) by the absence of GABA and glycine immunoreactivity and possession of an axon and a large soma in the RGC layer. Strong TRPV4 signal was concentrated in medium to large somas of RGCs, and some TRPV4 signal was found in BCs (including PKCα-positive rod BCs), as well as the end feet, soma and outer processes of Mȕller cells. TRPV4 immunoreactivity quantified by the pixel intensity histogram revealed a high-intensity component for the plexiform layers, a low-intensity component for the soma layers of ACs and Mȕller cells, and both components in the soma layers of RGCs and BCs. In large RGCs, TRPV4 agonists 4α-phorbol 12,13 didecanoate (4αPDD) and GSK1016790A reversibly enhanced the spontaneous firing and shortened the delay of voltage-gated Na^+^ (Nav) currents under current-clamp conditions, and under voltage-clamp conditions, 4αPDD largely reversibly increased the amplitude and frequency of spontaneous excitatory postsynaptic currents. In BCs, changes in the membrane tension induced by either applying pressure or releasing the pressure both activated a transient cation current, which reversed at ~ −10 mV and was enhanced by heating from 24 °C to 30 °C. The pressure for the half-maximal effect was ~18 mmHg. These data indicate that functional TRPV4 channels are variably expressed in primate RGCs and BCs, possibly contributing to pressure-related changes in RGCs in glaucoma.

## Introduction

Glaucoma is a retinal disease, characterized by the axon injury and soma loss of retinal ganglion cells (RGCs). It is a leading cause of blindness worldwide^1^, but its exact pathological mechanism is still uncertain. Glaucoma is involved with multiple risk factors, such as biochemical cascades, genetic defects, apoptotic cell death, glutamate excitotoxicity, free radical injury, mitochondria damage, glial activation, etc., while the elevation of the intraocular pressure (IOP) (either the mean level or the fluctuation)^2,3^ is a significant risk factor^1^. Although RGCs are intrinsically susceptible to IOP elevation, how elevated IOP damages RGCs has been debated. Neurons express ion channels in the plasma membrane, and some channels can be directly opened by force^4^ and are known as mechano-gated or mechanosensitive channels (MSCs). MSCs have been found in multiple types of retinal cells and postulated to contribute to glaucoma retinopathy^5,6^, one of which is the transient receptor potential channel (TRP) vanilloid 4 (TRPV4)^7,8^. Mutations in TRPV4 have been linked to axonal neuropathies in patients^9^, but the function of TRPV4 in the primate retina has not been studied.

MSCs are important for eukaryotic cells to balance osmotic and mechanical pressures across the plasma membrane. Although MSCs are typically adaptive to sustained mechanical stimuli^10^, TRPs do respond to transient signals (e.g. changes in mechanical force and light intensity). Circulation of the aqueous humor acts to stabilize IOP, yet, IOP still fluctuates to some extent. It shows 2–3 pulses per second in primates under physiological conditions^11^, and the amplitude is larger under higher IOP levels. In addition, retinal neurons may be stretched in childhood glaucoma (also known as buphthalmos). In chronic glaucoma, the optic disc cupping may stretch RGC axons there (e.g. for ~70–646 μm, derived from^12,13^). Therefore, MSCs can possibly be activated by both physiological and pathological IOP. Thus, it is essential to determine the effect of TRPV4 activation on activities of RGCs and other retinal neurons.

TRPs include seven subfamilies, namely TRPC (canonical), TRPV, TRPM (melastatin), TRPN (NOMPC), TRPA (ANKTM1), TRPP (polycystin) and TRPML (mucolipin)^14,15^. TRPs share the common feature of six transmembrane domains, various degrees of sequence similarity, and permeability to cations. The cation permeability (P) is usually indicated by the PCa/PNa ratio, which for TRPV1-6 (the six members of TRPV subfamily) is 3.8–9.6, 3, 2.8, 6, >100 and >100, respectively. The cation conductance allows TRPs to mediate membrane depolarization and Ca^2+^ influxes, which are known to be associated with neuronal excitotoxity. TRPs are variably modulated by temperature, osmolality, membrane tension, phorbol esters and G-protein-mediated regulation^16^, which allows identification of TRPV4. TRPV4 opens by pressure^17^, membrane stretch^18^, warm temperature and specific pharmacological agonists like GSK1016790A (GSK) and 4αPDD^15,19^. TRPV4, TRPM8, and TRPV3 work at similar temperatures. However, TRPV4 is a warm sensor activated at 27 °C^14,20^, while TRPM8 is a cold sensor and TRPV3 is a heat sensor activated at 23–28 °C and ≥33 °C, respectively. This study used the pressure sensitivity, thermosensitivity, specific pharmacological modulators, the reversal potential of TRPV4-mediated currents and immunolabeling to identify TRPV4 channels in primate RGCs and BCs.

Recently, morphological studies in glaucoma models have indicated that RGCs lose excitatory synapses while the axons are not lost^21^, and a functional study^22^ showed that IOP elevation reduces RGC light sensitivity by disrupting BC-RGC and BC-AII amacrine cell (AC) synaptic signals prior to retinal histological changes. RGCs receive excitatory glutamatergic synapses from BCs^23^. The b-wave of the electroretinogram (ERG) primarily reflects the function of BCs, and its amplitude and kinetics have showed changes in glaucoma retinas^24^. TRPV4 immunoreactivity has been observed in RGCs and the plexiform layers in the rat^25^ and porcine^8^ retinas. TRPV4 was located in mouse RGC dendrites, somas and axon bundles in the retina, optic nerve head and laminar region of the optic nerve, as well as in Müller cells^7,26^. TRPV4 immunoreactivity in the outer plexiform layer (OPL)^7,8^ displayed a horizontal expression pattern, so neuronal processes there are to be excluded to express TRPV4.

The structure and function of the primate retina do not fully resemble other mammalian retinas. For instance, visual signals converge into RGCs in different ways^27^, and the function of TRPV4 has not been examined in primate RGCs and BCs before. This study provides morphological and physiological evidence for the expression of functional mechanosensitive TRPV4 in the primate retina. Our data indicate that both RGCs and BCs are mechanically sensitive.

## Materials and methods

### Animals

This work used isolated retinal tissues from both macaques (*Macaca mulatta*) and baboons (*Papio cynocephalus anubis*). All procedures were carried out in strict accordance with the recommendations in the Guide for the Care and Use of Laboratory Animals of the National Institutes of Health and ARVO Statement for the Use of Animals in Ophthalmic and Vision Research. Isolated retinas used in this study were from third-party sources. Eyes were enucleated in ambient light illumination within 10 minutes after the animal had been overdosed with sodium pentobarbital (50–100 mg/kg, IV) at the conclusion of experiments that did not involve the eyes. The enucleated eyes were hemisected and then transported to our laboratory in oxygenated Ames medium (Sigma, St. Louis, MO) at room temperature. Eyecups with attached retinas were incubated in oxygenated Ames medium for 3–5 h^28^ in room temperature in ambient light illumination before experiments. 16 retinas mostly from 7 to 14-year-old animals were tested, focusing on the mid-peripheral and peripheral retina. Retinas were cut into 3 × 3 mm^2^ pieces, some of which were used for the immunocytochemistry and others for electrophysiological recording and the morphological study of recorded cells. The pieces for recording light responses were incubated in darkness for 1–2 h before the experiment. Each drug was tested two to three times for each cell to confirm the effect, and the current and voltage responses of a cell to a stimulus or drug were repetitively recorded for at least three trials.

### Whole-cell current-clamp, voltage-clamp, and loose patch recording

We recorded spontaneous action potentials under both loose-patch and current-clamp modes from 15 retinas. Only the peripheral retina (>7 mm away from the fovea) was selected for the recording, and each cell was recorded from a different retina. We also performed a systematic voltage-clamp analysis on spontaneous postsynaptic currents (PSCs) and light-evoked currents in RGCs. The excitatory and inhibitory PSCs were separated by holding the membrane potential to the cation or chloride equilibrium potential (E_C_ and E_Cl_, respectively), so that BC contributions to RGC light responses (cation currents, ΔI_C_, recorded at E_Cl_ ≈−60 mV) and contributions of amacrine cells (ACs) to RGC light responses (chloride currents, ΔI_Cl_, recorded at E_C_ ≈0 mV) could be separately studied^29–31^. This approach also allows us to separately record the effect of TRPV4 modulators on RGC spontaneous excitatory postsynaptic currents (sEPSCs, recorded at E_Cl_) mediated by BC synapses^29^ and spontaneous inhibitory postsynaptic currents (sIPSCs, at E_C_) mediated by AC synapses^30,31^. Another advantage of this approach is that individual RGCs can be filled with LY and/or NB during recording for the morphological identification of RGCs.

Whole-cell patch-clamp and loose-patch recordings of RGCs used flat-mounted retinal preparations. The sclera was removed, and the isolated retina was mounted to the bottom of the recording chamber with the RGC layer (GCL) up for recording. BCs were recorded from living retinal slices. A piece of the isolated retina was mounted to the bottom of the recording chamber and cut into 200–300-μm-thick slices with a home-made slicer. Each slice was remounted by turning 90 degrees to reveal the layers of the retina for recording. The preparation of living retinal slices essentially followed previous publications^22^. BCs locating in the first soma row of the inner nuclear layer with vertical oval-shaped somas were recorded and confirmed to be BCs after recording by their typical bipolar morphology^22^ (also see below). Procedures for recording light responses were performed under infrared illumination with dual-unit Nitemare (BE Meyers, Redmond, WA) infrared scopes. Whole-cell patch-clamp and loose-patch recording essentially followed the procedures reported in previous publications^22,32^. Oxygenated Ames solution (adjusted to pH 7.3) was introduced continuously to the recording chamber. A photostimulator was used to deliver light spots (of diameter 600–1200 μm) to the retina via the epi-illuminator of the microscope. The intensity of unattenuated (log I = 0) 500 nm light was 1.4 × 10^6^photons μm^−2^ s^−1^. Recordings were performed with an Axopatch 700B amplifier, a DigiData 1322A interface and pClamp software v9.2 (Axon Instruments, Foster City, CA). Recording pipettes had a tip diameter of 0.3–0.5 μm and the tip resistance of 5–8 MΩ, and they were filled with an internal solution containing 118 mM K gluconate, 10 KCl, 10 mM EGTA, 0.5 mM CaCl_2_, 1 mM MgCl_2_, 4 mM ATP, 0.3 mM GTP, 10 mM HEPEs, and 0.08 % LY (and/or 2% of neurobiotin (NB), Vector Laboratories, Burlingame, CA), adjusted to pH 7.2 with KOH. E_Cl_, with this internal solution, was −61 mV. For recording pressure-induced non-selective cation currents mediated by TRPs, K^+^ in the internal solution was replaced by Cs^+^^33^ to block K^+^ channels. The liquid junction potential at the tip of the patch electrode was compensated prior to seal formation with pClamp software. Drugs were dissolved in Ames mediums and applied in the bath. Specific TRPV4 agonists 4α-phorbol 12,13 didecanoate (4αPDD) and GSK1016790A (GSK), a general mechanosensitive channel blocker Ruthenium red (RR) (Tocris, Bristol, UK)^34^, as well as other chemicals, were purchased from Sigma-Aldrich unless stated otherwise.

A temperature control unit (TC 324B, Warner Instruments, CT) was used to control and monitor the temperature of the medium in the recording chamber. It was connected to the DigiData1322A to record the temperature of the medium. The medium was maintained at 34 °C for experiments in RGCs. Cold Ames medium (4 °C) was heated to different temperatures by the control unit to test the effect on BCs.

Positive and negative pressure (10–63 mmHg) steps were applied to cells during recording by altering the pressure inside the recording pipette after forming the giga-ohm seal (extracellularly) and after breaking through the membrane (intracellularly). The pressure was calibrated by a digital manometer DM8215 (Cole-Parmer, Vernon Hills, IL) with a resolution of 0.57 mmHg^35^. In some well-studied mechano-gated channels^36^, the convex membrane deformation facilitates the opening of mechanosensitive channels. The current and voltage responses of the recorded cell to pressure steps were recorded simultaneously with the temperature signals with a temporal resolution of 1–10 kHz.

After recording, retinas with LY- and /or NB-filled cells were fixed, stained with Cy3-, Cy5-, or Alexa Fluor 488-conjugated streptavidin (1:200, Jackson ImmunoResearch) and observed with confocal microscopes for the morphological identification of cell types. Then the flat-mount retinas with recorded RGCs were further sliced (see below) and observed again to accurately locate neuronal processes in the IPL^32^.

### Primary and secondary antibodies

Polyclonal rabbit anti-TRPV4 (LS-C135, 1:200; LS-A8583 1:200 and LS-C94498 1:100)^7^ was purchased from LifeSpan Biosciences, Inc (Seatle, WA). LS-C94498 was raised against a synthetic peptide from the cytoplasmic domain (aa100–150) of mouse TRPV4 conjugated to an immunogenic carrier protein. LS-A8583 targets a synthetic 20-amino acid peptide from the internal region of human TRPV4, and LS-C135 was raised against rat TRPV4 (Q9ERZ8, aa853–871, peptide immunogen sequence: CDGHQQGYAPKWRAEDAPL). In our hands, LS-C135 provided the best signal-to-noise ratio in the primate retina. The specificity of LS-A8583 and LS-C94498 for labeling retinal TRPV4 has been confirmed in TRPV4 knockout mice^7^, and LS-C135 and LS-A8583 provided similar labeling patterns (see Results). These data support the specificity of these antibodies. Other primary antibodies included in this study have also been used in previous reports, including polyclonal guinea pig anti-GABA (1:1000, AB175; Chemicon, Temecula, CA)^37^ and rat anti-glycine antiserum (1:1000, a generous gift from Dr. David Pow, University of Queensland, Brisbane, QLD, Australia)^38^. Protein Kinase-C alpha (PKCα) is a classic marker for rod BCs^39^. The anti-PKCα antibody from Sigma (P4334, 1:1000, rabbit, polyclonal) has been tested in immunoblotting in rat brain extract, and it recognized a heavy band at ~ 76 kDa and a very weak band at 40 kDa, while the predicted molecular weight of the PKCα was 76–93 kDa. The staining was specifically inhibited by PKCα immunizing peptide (659–672). The monoclonal anti-PKCα antibody from BD transduction (610107, Clone 3/PKCα (RUO), 1:200, mouse) identified a single band at 82 kDa from a rat cerebrum lysate. Monoclonal mouse anti-glutamine synthetase (GS) (1: 1000, clone 6, BD Transduction Laboratories, Palo Alto, CA) was used to identify Mȕller cells^40^. The antibody was raised against the human glutamine synthetase aa 1–373 and recognized a band at ~45 kDa, consistent with the predicted molecular weight of GS.

The specificity of these primary antibodies has been demonstrated in the previous studies, and their staining patterns in our results were similar to the previous reports. Controls were also processed with blocking peptides or without primary antibodies. All controls did not show positive results.

### Immunocytochemistry

Retinal tissues from 16 retinas were fixed with 4% paraformaldehyde in phosphate buffer (pH 7.4) for 1–2 h at 4 °C. They were then blocked with 10% donkey serum (Jackson ImmunoResearch, West Grove, PA) in TBS ((D-PBS with 0.5% Triton X-100 (Sigma-Aldrich) and 0.1% NaN3 (Sigma-Aldrich)) for 2 h at room temperature or at 4 °C overnight to reduce nonspecific labeling. A small piece of the retina was embedded in low gel-point agarose (Sigma-Aldrich) and trimmed into a 10 × 10 × 10 mm^3^ block. The block was glued onto a specimen chamber mounted on a vibratome (Pelco 102, 1000 Plus; Ted Pella, Inc., Redding, CA) and subsequently cut into 40-μm-thick vertical sections in PBS solution^40^. For staining, retinal tissues were incubated in primary antibodies in the presence of 3% donkey serum-TBS for 3 to 5 days at 4 °C. After several rinses, they were transferred into Cy3-, Cy5-, or Alexa Fluor 488-conjugated streptavidin (1:200, Jackson ImmunoResearch), with Cy3- and/or Cy5-conjugated secondary antibodies (1:200, Jackson ImmunoResearch) and/or Alexa Fluor 488-conjugated secondary antibodies (1:200, Molecular Probes, Eugene, OR), in 3% normal donkey serum-TBS solution at 4 °C overnight. A nuclear dye, TO-PRO-3 (0.5 μL/mL, Molecular Probes, Eugene, Oregon) was used with the secondary antibody to visualize nuclei in retinas. After extensive rinsing, retinal preparations were cover-slipped. Two small pieces of filter paper (180-μm thick, MF-membrane filters; Millipore, Billerica, MA) were mounted beside flat-mount retinas to prevent them from being over-flattened.

### Confocal microscopy

Zeiss confocal microscopes (LSM 510 and LSM 800, Carl Zeiss, Germany) and imaging software were used for morphological observation. Recorded cells were observed with a 40× water immersion lens (for RGCs) and 40× and 60× oil lenses (all cells). A series of optical sections were made over each recorded cell, including the soma and all processes, for better morphological identification. The entire dendritic arbor was revealed by the *x*-*y* view of the reconstructed 3D image of the cell. The dendritic ramification pattern in the inner plexiform layer (IPL) was revealed either in retinal slices or by the *y*-*z* and *x*-*z* views of the reconstructed 3D image of the recorded cell. Previously established methods were used to survey RGC density in the flat-mounted retinas^40,41^ and the soma size^40^ of TRPV4-positive RGCs. Confocal micrographs were further processed with Photoshop (Adobe Systems Incorporated, San Jose, CA) software, typically by enhancing the contrast and selecting color channels with better visibility for light-adapted human eyes. In this paper, some confocal micrographs are presented with a white background, which was achieved simply by inverting the image of a black background with Photoshop software. The level at which dendritic processes stratified in the IPL was described by the distance from the processes to the distal margin (0%) of the IPL. RGCs were counted in flat-mount retinas with confocal and Photoshop software. Immunolabeled retinas were generally examined with a vertical resolution of 0.4–1.2 μm under regular line-scan and frame-scan modes and further examined with confocal Airyscan protocol and software with a pixel size of 30 nm. The Airyscan images were displayed by the 3D surface profile reconstructed from a series of optical sections obtained with a step of 180 nm. The immunoreactivity was quantified by the pixel intensity histogram in original confocal images without any modification.

### Statistical analysis

Data were analyzed by Sigmaplot software (v12, Systat, Point Richmond, CA), Clampfit (v10.3 and v9.2, Axon Instruments, Foster City, CA), and Microsoft Excel and presented as *mean* ± *s.e*. Two-tail Student *t-test* was used for analyzing statistical significance between paired data groups. The α level to reject the null hypothesis was 0.05. The relationship of the membrane potential (V) and the delay time (T) of Na^+^ currents mediated by voltage-gated Na^+^ channels (I_Na_) was well fit by a standard exponential function $f\left( V \right) = \mathop {\sum }\limits_{i = 1}^n T_ie^{ - V/_i} + C$. The pressure (P)-response curves were well fit by an exponential cumulative distribution function $f\left( P \right) = \mathop {\sum }\limits_{i = 1}^n R_i(1 - e^{ - P/_i}) + C$, where *R* was the amplitude of normalized responses. The histograms of pixel intensity (I) were well fit by a Gaussian function $f\left( I \right) = ae^{\left[ { - 0.5\left( {\frac{{I - I_0}}{b}} \right)^2} \right]}$, where a is the maximum frequency and I_0_ is the peak intensity. Clampfit and Sigmaplot software was used for finding the best fitting functions.

## Results

### TRPV4 was most intensively expressed in large- to medium-sized RGC somas

**Fig. 1:**
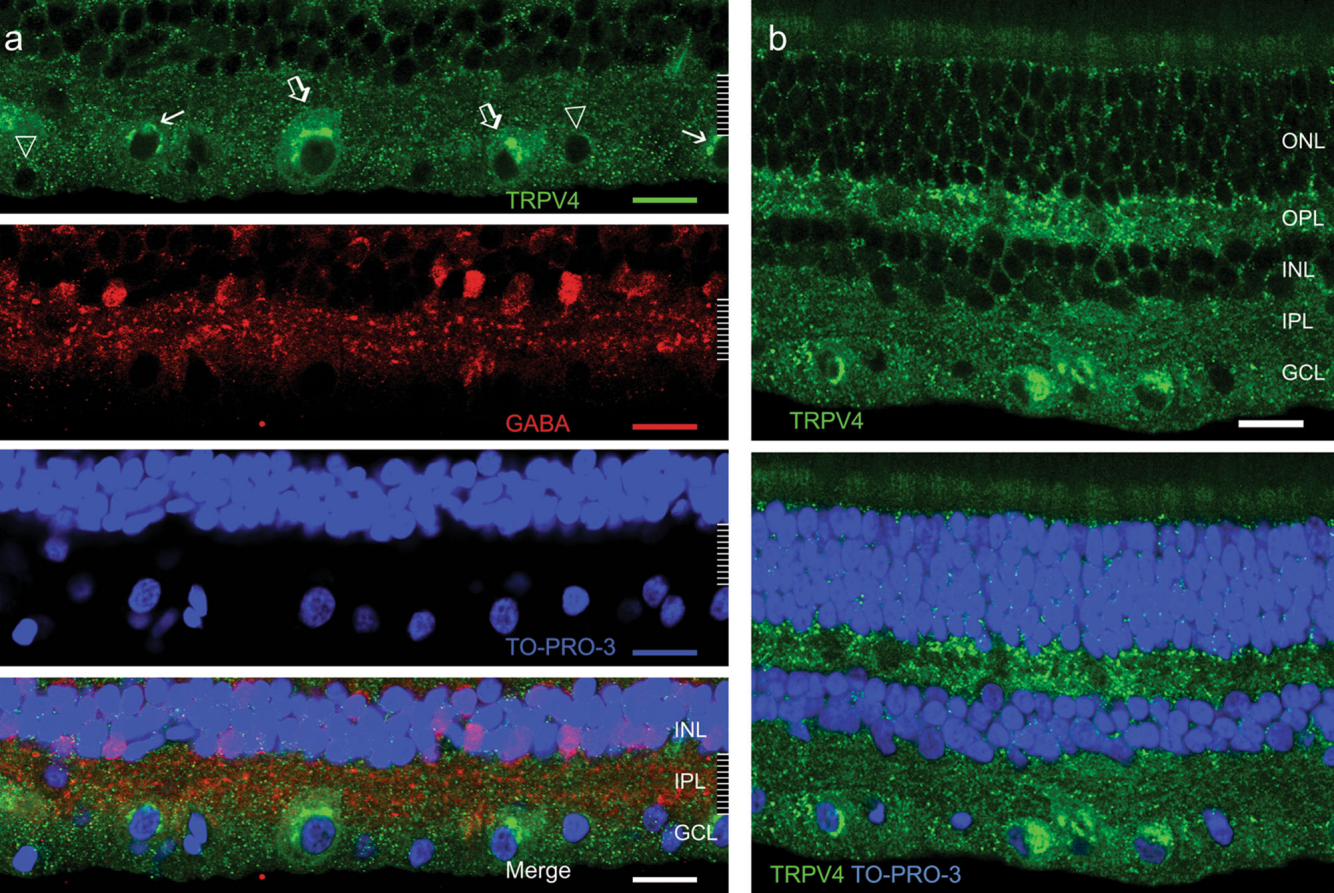
TRPV4 expression in the primate retina. Confocal micrographs of retinal slices are double or triple-labeled for TRPV4 (green, LS-C135), GABA (red, **a**) and TO-PRO-3 (blue). TRPV4 signals appear as puncta, and large and medium RGCs (open arrow, **a**) in the ganglion cell layer (GCL) negative for GABA are the most intensively labeled. In these RGCs, TRPV4 puncta are larger and denser in the cytosol and absent inside the nucleus. Smaller RGCs in the GCL that are negative for GABA are moderately (arrow, **a**) to weakly (triangle, **a**) positive for TRPV4. TRPV4 signals show a higher density in the outer and inner plexiform layers (OPL and IPL, respectively, **b)** than in the inner and outer nuclear layers (INL and ONL, respectively). In nuclear layers, TRPV4 signals are sparse and often surround cell bodies (**b**). Scale bars are 20 μm

We examined TRPV4 immunoreactivity in 8 retinas with three TRPV4 antibodies. RGCs were differentiated from ACs by the absence of clear GABA and glycine immunoreactivities. TRPV4 immunoreactivity appeared as small to large puncta (Fig. 1). Small TRPV4 puncta revealed soma boundaries of some RGCs and plexiform layers, indicating the expression in the neuronal plasma membrane. Clusters of large TRPV4 puncta were observed in the cytosol of large and medium somas of RGCs (≥15 μm in diameter)^42,43^, particularly in the perinuclear region, presumably in the rough endoplasmic reticulum where proteins are synthesized. The specificity of LS-A8583 and LS-C94498 has been previously confirmed in the TRPV4 knockout mouse^7^. LS-C135 and LS-A8583 provided similar labeling patterns. Smaller somas in the GCL were generally more weakly labeled compared with larger ones (Fig. 1). Brightly labeled RGC somas were distributed sparsely in the retina, and their density was estimated to be 77 ± 11cells/mm^2^ (*n* = 2 retinal preparations) in the peripheral retina. RGC somas possessed only a few small TRPV4 immunoreactive puncta were not counted due to the low visibility.

### The expression of TRPV4 in other retinal layers

**Fig. 2:**
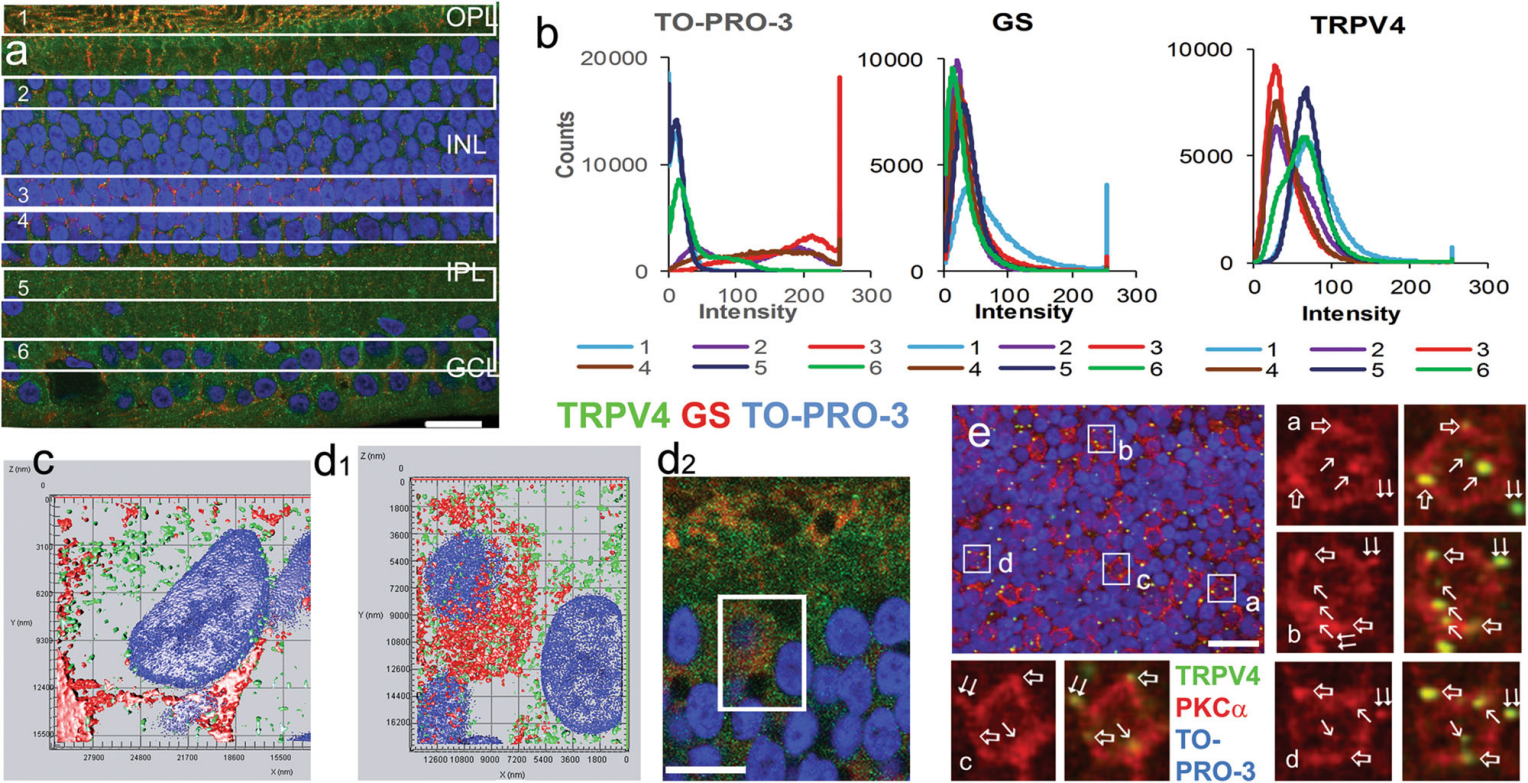
TRPV4 immunoreactivity in retinal neurons and Mȕller cells. Monkey retinal slices (**a**–**d**) were labeled for TRPV4 (LS-C135), glutamine synthetase (GS) and TO-PRO-3. **b** shows pixel histograms of TRPV4 immunoreactivity in 6 same sized retinal zones in **a** (1-the outer plexiform layer, 2-the bipolar cell soma layer (BCL), 3-the Mȕller cell soma layer (MCL), 4-the amacrine soma layer (ACL), 5- the inner plexiform layer and 6-the RGC soma layer (GCL). GS-positive somas are primarily located in Zone 3, where the linear density of TO-PRO-3 labeled nuclei is higher than that in Zone 2 and 4 (ratio: 1.8: 1.2: 1) (**a** and **b**). TRPV4 pixel histograms generally fall into two groups, one for those from Zone 1, 5, and 6 and the other for those from Zone 2, 3, and 4 (**b**). **c** and **d**_1_ are the surface profile of 3D projections of 0.9 μm-thick blocks in the GCL (**c**) and BCL (**d**_1_), and TRPV4 puncta are not fully colocalized with GS. **d**_1_ displays the inset of **d**_2_. In **e**, a flat-mount monkey retina was labeled for TRPV4 (LS-C94498, green), PKCα (red), and TO-PRO-3 (blue). The confocal micrograph shows the optical section of the BCL, where TRPV4 puncta are colocalized with PKCα inside the somas (arrow), somatic membrane (open arrow) and dendrites (double arrow) of rod bipolar cells (RBCs). TO-PRO-3 labels nuclei, Scale bars are 20 μm

The intensity of TRPV4 immunoreactivity was higher in the GCL and the inner and outer plexiform layers (IPL and OPL, respectively) compared with the inner and outer nuclear layers (INL and ONL, respectively), and TRPV4 was not fully colocalized with GS (Fig. 2). GS-labeled somas of Mȕller cells were primarily arranged in a layer (MCL) at ~66% of the INL depth (with 0% representing the outer border) resembling previous findings^40,44^, and the layer was also identifiable by the higher linear density of TO-PRO-3-labeled nuclei compared to that in the upper (the BC soma layer, BCL) and the lower half (the AC soma layer, ACL) of the INL (ratio: 1.8: 1.2: 1) (Fig. 2a, b). TRPV4 immunoreactivity was observed in Mȕller cells’ processes in the OPL (Fig. 2a and d_2_), somas in the INL (Fig. 2d), and end feet in the GCL (Fig. 2c), while some TRPV4 puncta in the GCL (Fig. 2c) and BCL (Fig. 2d) were not colocalized with GS. Some TRPV4 puncta were colocalized with PKCα in somas and dendrites of rod BCs (RBCs) (Fig. 2e). Intensity histograms of TRPV4 pixels (Fig. 2b) were well fit to a Gaussian function (see method) (all *p* *<* 0.0001), consisting of either a high-intensity (OPL and IPL; *b*: 17.4–24.4; *I*_*0*_: 67.5–73.4) or a low-intensity (MCL and ACL; *b*: 16.8–19.9; *I*_*0*_: 31.6–36.1) component or both (GCL and BCL). The GCL histogram (*b*: 25.5; *I*_*0*_: 61.7) and BCL histogram (*b*: 27.5; *I*_*0*_: 41.8) contained both components, but the former showed higher peak intensity *I*_*0*_. Histograms from the BCL, ACL, and MCL were similar, while that of the MCL showed the highest *a* value (Fig. 2b). The data indicate that TRPV4 is expressed in neurons in the GCL and BCL.

### Activating TRPV4 enhanced the firing rate, sEPSC amplitude and frequency, and the membrane excitability of parasol RGCs

**Fig. 3:**
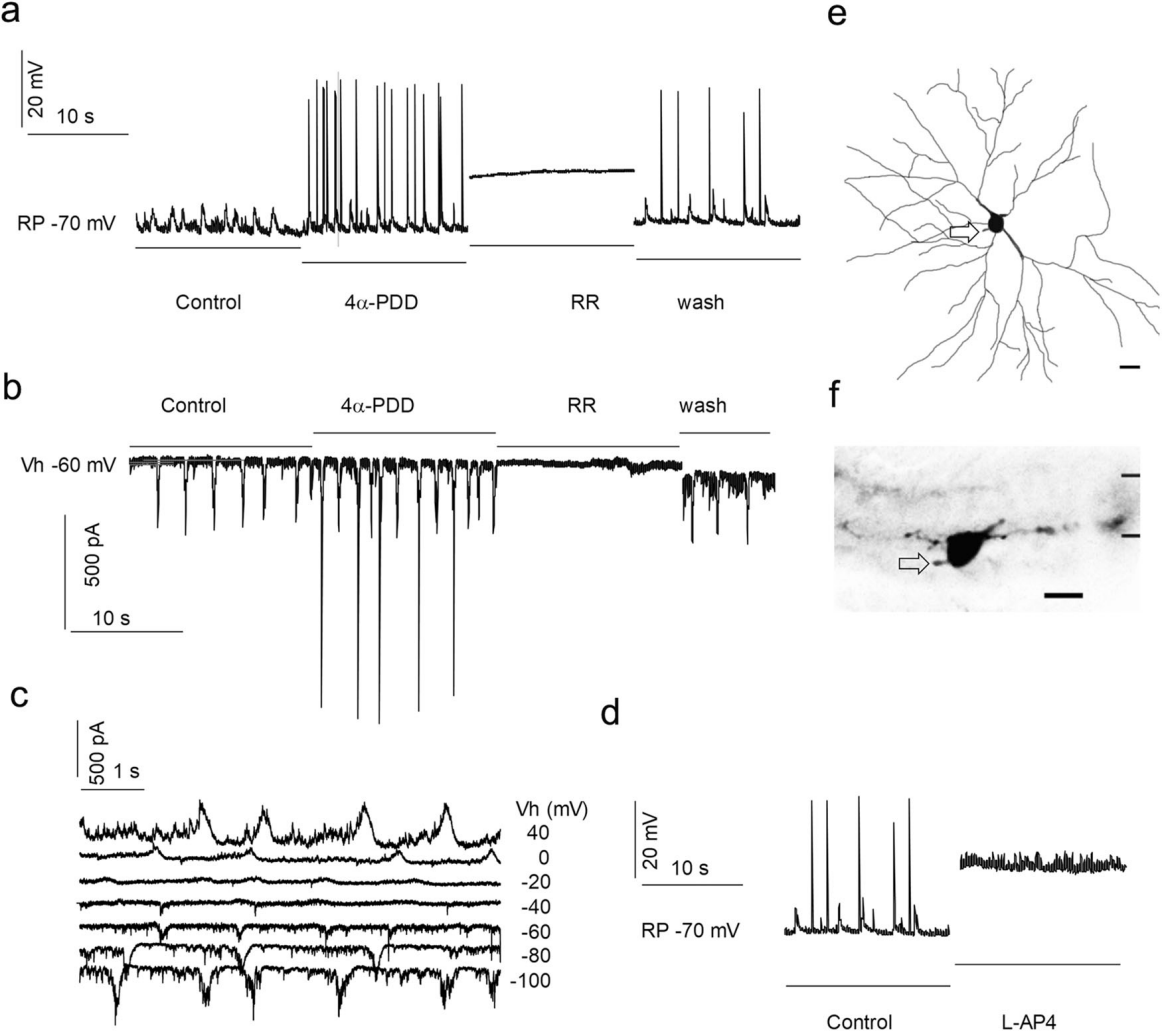
The activation of TRPV4 enhances the amplitude and frequency of spontaneous excitatory postsynaptic currents (sEPSCs)in RGCs. A RGC was recorded under whole-cell current-clamp (**a**, **d**) (holding current I = 0) for action potentials and voltage-clamp (**b** and **c**) modes for spontaneous postsynaptic currents (sPSCs) from a flat mount retina. sEPSCs were recorded at the chloride equilibrium potential (E_Cl_, −61 mV). The bath application of TRPV4 agonist 4αPDD (0.4 μM, **a**, **b**) evokes firing of action potentials (**a**) and an increase in the frequency and amplitude of sEPSCs (**b**). These effects were reversibly abolished by a general MSC blocker ruthenium red (RR) (5 μM). sPSCs (**c**) reverse near −20 mV and action potentials and spontaneous postsynaptic potentials are abolished by mGluR6 agonist L-AP4 (**d**), demonstrating that the activities are dominated by chemical synapses from ON bipolar cells. The cell was identified as an ON cell by neurobiotin labeling. The cell morphology revealed from the flat-mount retina (**e**) shows a soma of 27 μm in diameter and a dendritic field of 356 × 267 μm. The dendrites observed from retinal slices (**f**) ramify around 70% of the IPL depth. In **e** and **f**, arrows show the axon, and scale bars are 20 μm. Vh-holding potential; RP-resting potential

**Fig. 4:**
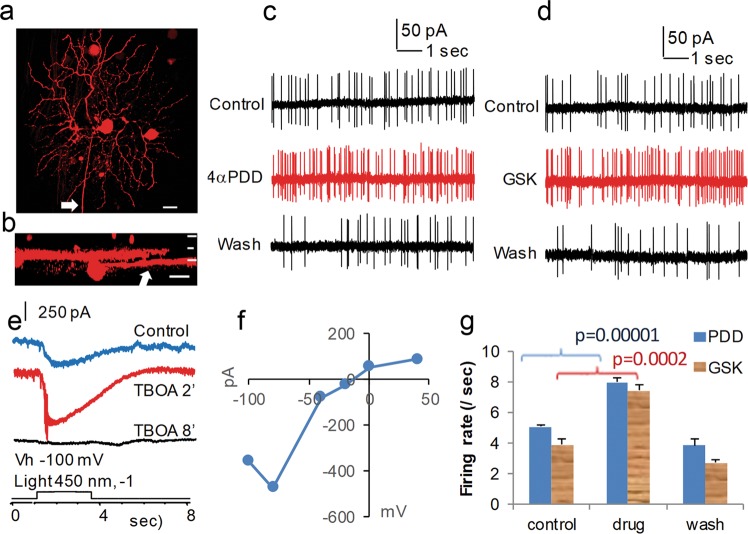
Opening TRPV4 enhances the spontaneous firing in parasol ganglion cells. **a** to **f** show an RGC, which was recorded for action potentials under loose-patch mode (**c** and **d**) and for light-evoked currents under voltage-clamp mode (**e** and **f**) from a flat mount retina. The cell was filled with neurobiotin during recording. Confocal micrographs (**a** and **b**) morphologically identify the cell as an ON parasol cell. The x-y view (**a**) and y-z view (**b**) of the 3D reconstructed cell images reveal a soma of 25 μm in diameter and a dendritic arbor of 254 × 218 μm ramified round 65% of the IPL depth. Current responses evoked by the light steps of a duration of 2.5 s reverse near −15 mV (**e** and **f**) and are inward cation currents at E_Cl_ (−61 mV), and the light-evoked current (**e**) was enhanced by 250 μM TBOA (a glutamate transporter inhibitor) after 2 minutes of bath application of the drug and fully abolished after 8 minutes, which indicate that the activities are dominated by ON bipolar cell inputs. TRPV4 agonists 4αPDD 0.4 μM (**c** and **g**) and GSK 0.4 μM (**d** and **g**) applied in the bath show similar effects on RGCs (**g**), which significantly and reversibly increase the spontaneous firing rate (**g**, *n* = 5 experiments/cells, two-tail *t-test, p* < 0.001 for both drugs). In **a** and **b**, the arrow shows the axon and scale bars are 20 μm. Vh-holding potential

For electrophysiological recordings, current responses of cells were recorded under voltage-clamp conditions, voltage responses and action potentials under current-clamp conditions, and spikes under loose patch conditions. To understand the function of retinal TRPV4, we examined the effect of TRPV4 channel modulators on RGC spontaneous action potentials and sEPSCs (Figs. 3 and 4). Recorded RGCs were filled with neurobiotin (NB) and/or Lucifer yellow (LY) during patch-clamp recording. The morphology of each recorded cell was examined with confocal microscopy first in the flat-mount retina and then in vertical slices. Parasol RGCs were identified by their morphology and physiology.

TRPV4 channel agonists 4αPDD (≤2 μM) and GSK (≤1 μM) significantly enhanced the spontaneous firing rate of action potentials (Figs. 3 and 4) and the frequency and amplitude of sEPSCs (Fig. 3) in parasol RGCs (*n* = 5 cells). The frequency of events was increased ~2.1 times (*n* = 54 trials) and the amplitude of sEPSCs were ~2.3 times larger (*p* < 0.0001, *n* = 19 trials). These effects were reversibly abolished by a general MSC blocker ruthenium red (RR).

The spontaneous action potentials were abolished by mGluR6 agonist L-AP4 in ON cells (Fig. 3d). The reversal potential of spontaneous postsynaptic currents (sPSCs) (Fig. 3c) and light-evoked currents (Fig. 4f) were near 0 to −20 mV, which was closer to E_C_ (0 mV) than E_Cl_ (−61 mV). These results support the idea that activities of parasol RGCs are dominated by chemical synapses from BCs instead of ACs. sEPSCs were recorded at E_Cl_ (see Methods for details), separating the excitatory inputs (from BCs) from the inhibitory chloride currents (from ACs)^29,31^. In the CNS, it has been known for many years that the frequency of spontaneous events is due to presynaptic release properties^45,46^. Taken together, the data indicate that opening TRPV4 channels enhances spontaneous excitatory inputs from BCs to RGCs.

**Fig. 5:**
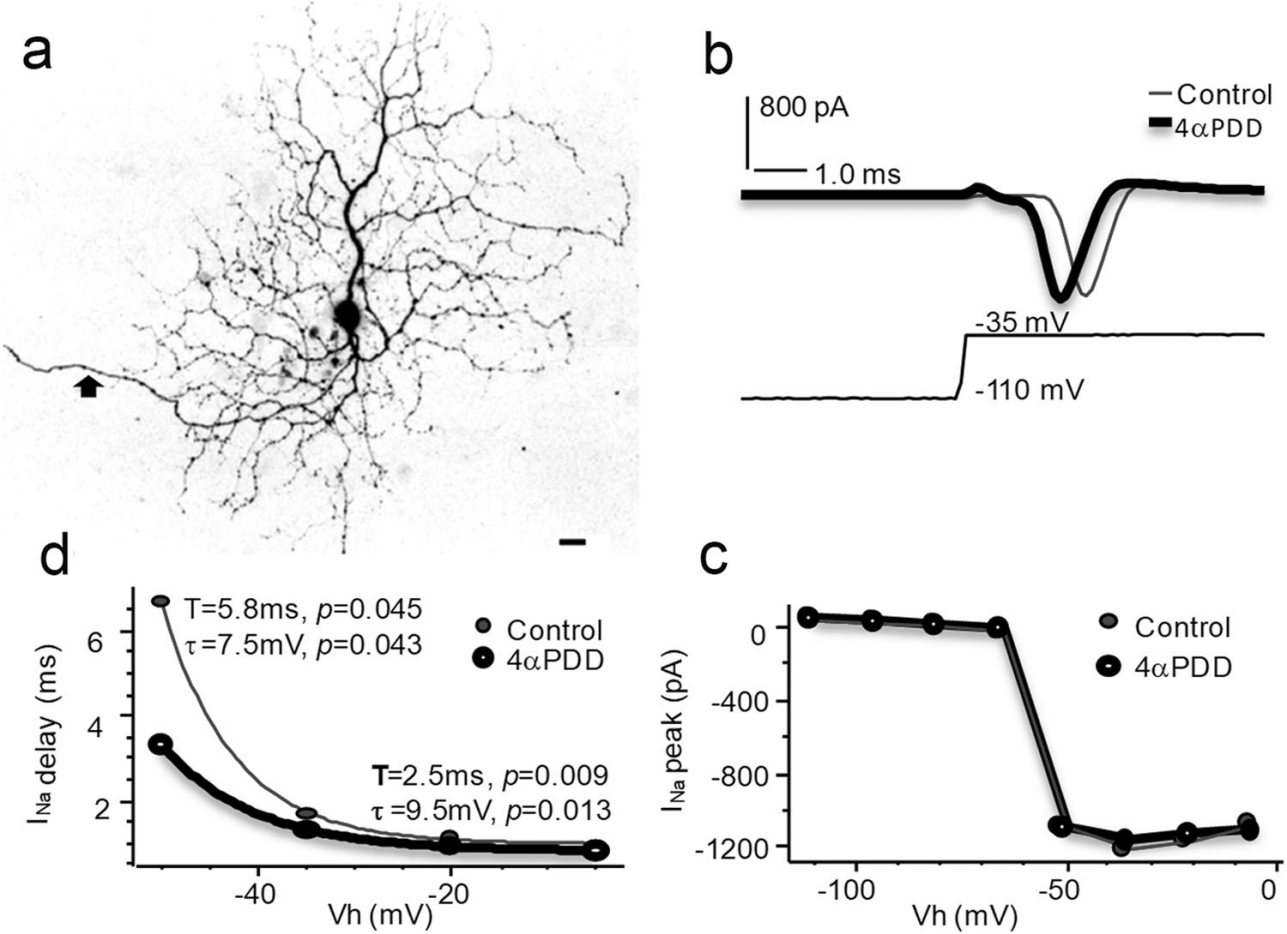
Activating TRPV4 enhances membrane excitability of parasol ganglion cells. Na currents (I_Na_) mediated by voltage-gated Na channels were recorded under whole-cell voltage-clamp mode from flat mount retinas. Electric pulses were used to hold the membrane potential from a baseline level of −110 mV (**b** and **c**) or −70 mV (**d**) to a series of Vh. The I_Na_ is activated at Vh ≈ −50 mV (**c**). The application of TRPV4 agonist 4αPDD 1 μM in the bath does not clearly alter the activation curve (**c**) or peak amplitude of I_Na_ (**b**), while the delay time (T) of I_Na_ is shortened for all suprathreshold stimuli (**d**). The relationship of T and Vh is significantly altered (p < 0.05 for both T and τ) (For definitions of τ see methods). In **a**, the arrow depicts the axon, and the scale bar is 20 μm. The chloride equilibrium potential is −61 mV. Vh-holding potential

We further studied the effect of TRPV4 agonists on Na^+^ currents (I_Na_) in parasol RGCs mediated by voltage-gated Na^+^ channels (Nav) (Fig. 5). I_Na_ was evoked under voltage-clamp conditions by depolarizing RGC membrane potentials from −110 or −70 mV with a step of 8–15 mV, which would not be significantly affected by BC and AC synapses. I_Na_ was activated at ~−50 mV (*n* = 5 cells), consistent with voltage-gated Na channels well documented in previous literature^47,48^. The peak amplitude, as well as the delay time of I_Na_, i.e. the time between the beginning of stimuli to the beginning of evoked inward I_Na_, was examined before and during bath application of drugs for 1–3 min. The data showed that the drug did not clearly alter the activation curve or the peak amplitude of I_Na_, but it shortened the delay time of I_Na_ evoked by all depolarizing pulses above the threshold (*p* < 0.05), which indicate that activating TRPV4 increases RGC membrane excitability.

### The pressure and temperature sensitivity of bipolar cells

**Fig. 6:**
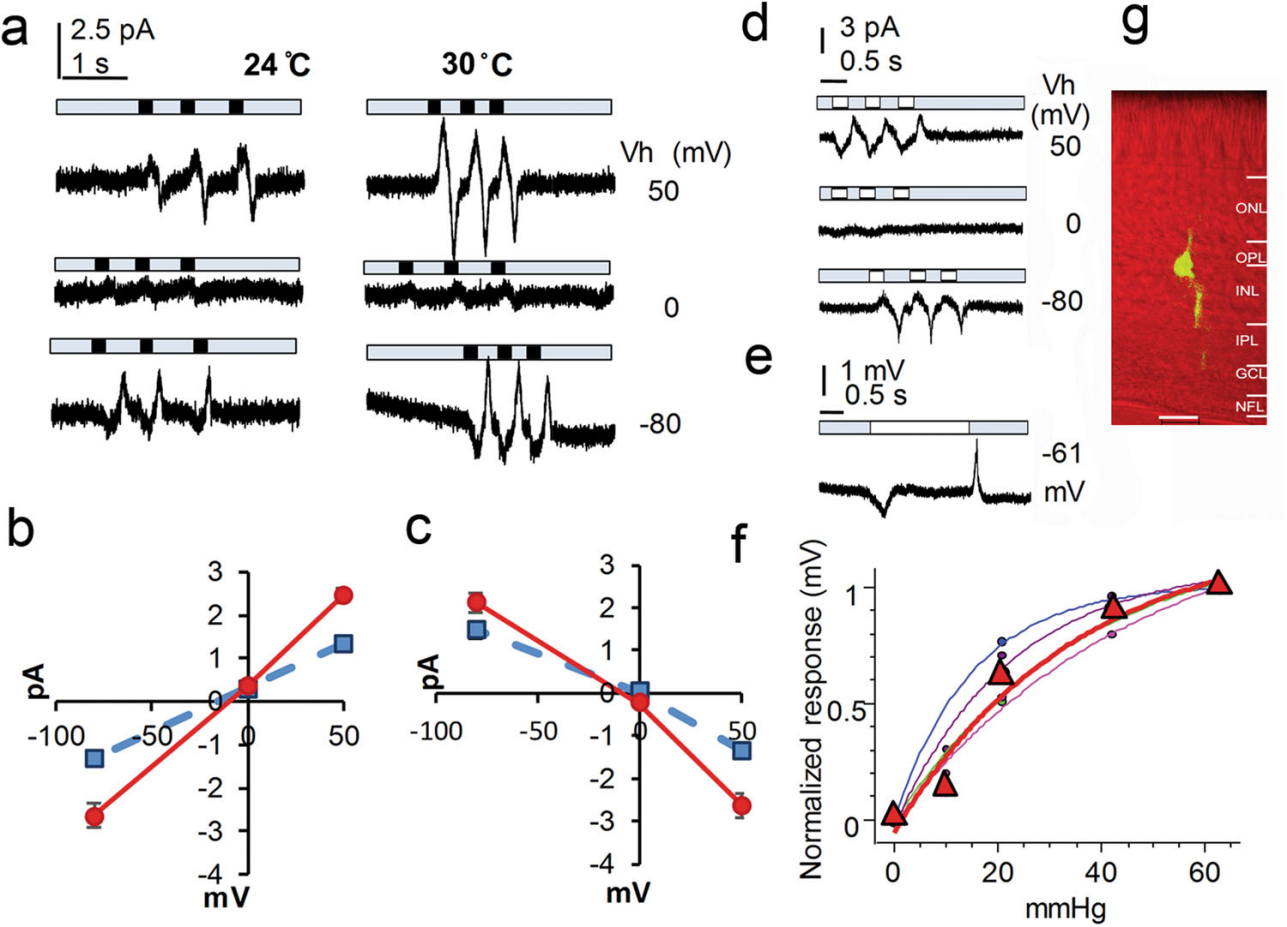
Pressure and temperature sensitivity of bipolar cells. The internal solution in the recording pipette contained Cs^+^ to block K^+^ channels. **a** shows *current* responses to brief pressure steps of 42 mmHg (black rectangles) applied to the intracellular side at holding potentials (Vh) of −80, 0 and 50 mV. The onset of pressure steps activates a cation conductance that reversed near −10 mV (**b**), and the offset reduces the cation conductance (**c**). Currents elicited by the positive (**a**) and negative (**d**, −42 mmHg, white rectangles) pressure steps show opposite polarities but the same reversal potential. Pressure-induced responses were largely enhanced upon heating from 24 °C (**b** and **c**, blue squares and dashed lines) to 30 °C (**b** and **c**, red dots and solid lines) (*p* < 0.001, *n* = 6 experiments/cells). Negative pressure of longer durations (**e**, −42 mmHg, white rectangles) applied to the intracellular side elicits transient *voltage* responses. The pressure - normalized response curves are well fit by an exponential cumulative distribution function (**f**, dots and fine lines) (*n* = 4 experiments/cells), and the mean is displayed in red (triangles and the thick line). **g**-confocal image of a recorded bipolar cell filled with Lucifer yellow (yellow) and presented against a Nomarski view (red) of the retinal slice. The scale bar in **g** is 20 μm

In retinal slices, we recorded pressure-induced responses in BCs with vertical oval somas located in the distal half of the inner nuclear layer (Fig. 6). The cells were filled with LY and/or NB during recording and identified as bipolar cells by a typical bipolar morphology with dendrites extending into the OPL and an axon descending to the IPL (Fig. 6). Pressure steps of a duration of 200–3000 ms evoked transient responses in BCs. Positive pressure applied to the intracellular side activated a cation conductance which reversed at ~ −10 mV, and releasing the pressure reduced the cation conductance. Negative pressures applied to the intracellular side induced responses of opposite polarities but the same reversal potential. The pressure-response curve was well fit by an exponential cumulative distribution function and τ was 34 mmHg (range: 15–46 mmHg) (For the definition of τ, see Methods). The mean pressure to evoke the half-maximal response was ~18 mmHg (range: 10–23 mmHg). Upon heating the bath from 24 °C to 30 °C, the amplitude of the pressure-induced responses increased by 40–100%.

## Discussion

### Parasol RGCs and other large RGCs in the primate retina intensively express TRPV4

We identified parasol RGCs by their soma size and dendritic morphology^49^. We also distinguished RGCs from ACs by their axons, large somas and the absence of the immunoreactivities to GABA and glycine. RGCs in the mouse^7^, rat^25^ and porcine^8^ retinas have been reported to express TRPV4, and our data are consistent with these previous studies. 4αPDD and GSK have been used to identify TRPV4 in the retina^7^ and elsewhere^15^. TRPV4 is a pressure-sensitive warm sensor, and our results showed that BCs responded to both pressure and warm temperature. Our immunocytochemical results, combined with others’ studies^7,8,25^ and our physiological data, support that TRPV4 was properly identified and it was intensively expressed in large RGCs.

### Opening TRPV4 channels excites both BCs and RGCs

The opening of TRPV4 induced by pharmacological agonists and hypotonicity is known to depolarize the neuronal membrane and increase the spontaneous firing rate in mouse RGCs^7^, but it has been unclear whether the effect is related to BCs. The IPL and OPL in mammalian retinas^7,8^ have been previously shown to express TRPV4, yet BCs have not been known to express TRPV4 before. Our results showed that RGCs, plexiform layers, and BCs express TRPV4. Additionally, this study is the first to show that opening TRPV4 enhances sEPSC frequency in RGCs and that BCs are mechanosensitive. The pressure for evoking the half-maximal response in BCs reported in this study is comparable to that for mechano-gated channels in other studies^36^.

Mȕller cells express TRPV4^50^ but make no synapses with retinal neurons^23^. They express the glutamate transporter GLAST^51^ to modulate neuronal activities. Glutamate transportation relies on the energy stored in the Na^+^ electrochemical gradient, and opening TRPV4 in cells causes Na^+^ influxes^14,26^. Therefore, opening TRPV4 in Mȕller cells would reduce glutamate removal near synapses. However, reducing glutamate removal by either acutely inhibiting^52^ or knocking out GLAST^53^ was found to *reduce* the ERG b-wave instead of exciting BCs. Data from BC/putative RGC pairs showed that reducing glutamate uptake in Mȕller cells did not alter the amplitude, time course, or frequency of sEPSCs in RGCs, though evoked EPSCs were elongated^54^. Recent studies further confirmed key distinctions between the synaptic vesicle fusion machineries that perform spontaneous versus evoked neurotransmitter release^46^. Moreover, mechanically stimulating Mȕller cells was found to inhibit RGCs^55^. Therefore, we feel that TRPV4 in Mȕller cells is not accountable for our physiological results in RGCs and BCs.

We applied pressure stimulation in individual BC somas. The mechanical sensitivity is, therefore, primarily attributed to ion channels located in BCs. Although multiple mechanically sensitive channels are thermosensitive, TRPV4 has unique thermosensitivity^14,15^ and it has not been found in photoreceptors or HCs^7,8,25,26^. Therefore, our physiological and morphological results together indicate that BCs are mechanically sensitive and express TRPV4. Our data, however, did not fully exclude other MSCs in BCs from contributing to the BC’s mechanical sensitivity. In the CNS, the frequency of spontaneous events is due to presynaptic release properties while the amplitude and shape of the response are largely attributed to postsynaptic changes in ionotropic receptor responses^45,46^. Thus, we think that the effect of 4aPDD on the frequency of sEPSCs in RGCs is accounted for by TRPV4 in BCs; and the effect of 4aPDD on the amplitude of sEPSCs in RGCs is primarily attributed to TRPV4 in RGCs. Further studies on isolated cells combined with pharmacological channel antagonists will likely better map the expression of mechanically sensitive channels in subtypes of retinal neurons.

In the peripheral nervous system, a few reports have shown that Na^+^ currents are sensitive to thermal and mechanical stimuli ^56–58^, and our data are consistent with these previous reports. Additionally, we further showed that opening TRPV4 in RGCs enhanced the membrane excitability.

### TRPV4 and BCs likely play some roles in glaucoma

Glaucoma retinopathy is highly correlated with IOP elevation and how RGCs are damaged is not clear^1^. Our results show that RGCs and BCs express TRPV4, opening TRPV4 excites RGCs, and BCs are mechanically sensitive. The results, in line with others’ findings^7,8^, suggest that TRPV4 and BCs may play some roles in glaucoma.

Glutamate excitotoxicity is an important mechanism underlying neuronal degenerative diseases in the CNS including glaucoma^59^, but triggers for excessive glutamate release in glaucoma have not yet been identified. TRPV4-mediated Ca^2+^ and Na^+^ influxes can possibly elicit glutamate release from BCs to RGCs^23^. This study is the first to reveal functional TRPV4 channels and mechanical sensitivity in BCs, providing a novel potential route for IOP elevation to enhance glutamate release.

RGCs receive convergent inputs from BCs and the convergence is more intensive in the peripheral retina than in the central retina^60^. Rods are the dominant photoreceptor types in the vast peripheral retinal region and rod BCs feed excitatory signals to RGCs. Therefore, we deduce that the extensive convergence of BC inputs would allow the low density of TRPV4 and pressure-induced small responses in BCs to more dramatically affect the function of RGCs in the mid-peripheral and peripheral retina than those in the central retina. Pressure-induced transient responses in BCs also suggest that TRPV4 could possibly respond to IOP fluctuation^11^. Our data together indicate that TRPV4 plays important roles in primate retinal BCs and RGCs, and RGCs and BCs are both mechanosensitive neurons.
